# The experiences of health workers using telehealth services for diabetes-related foot complications: a qualitative exploration

**DOI:** 10.1186/s13047-023-00645-9

**Published:** 2023-08-09

**Authors:** Kristin Graham, Christie Marie Siatis, Kate M. Gunn, Emilee Ong, Cathy Loughry, Neil McMillan, Robert Fitridge

**Affiliations:** 1 Allied Health and Human Performance, The University of South Australia, North Terrace, Adelaide, SA 5000 Australia; 2 Department of Podiatry, Central Adelaide Local Health Network, Adelaide, SA Australia; 3 Basil Hetzel Institute for Translational Health Research, Central Adelaide Local Health Network, Woodville South, SA Australia; 4 Adelaide Medical School, The University of Adelaide, Adelaide, SA 5000 Australia; 5 Vascular and Endovascular Surgery Service, Royal Adelaide Hospital, Adelaide, Australia

**Keywords:** Diabetes-related foot disease, Indigenous, Real-time video-based telehealth, Rural, Remote

## Abstract

**Background:**

Diabetes-related foot disease (DFD) accounts for up to 75% of lower-extremity amputations globally. Rural and remote communities are disproportionately affected by DFD. Telehealth has been advocated as a strategy to improve equity of access to health care in rural and remote communities. Current literature suggests that successful implementation of telehealth requires access to adequate reliable equipment, staff training, and support. A real-time video-based telehealth foot service (TFS) for delivering DFD management has recently been established in a Vascular Surgery and Podiatry clinic within a large South Australian metropolitan hospital. The purpose of this study was to gain insights into the experiences of rural and remote health professionals utilising the TFS, as this could be invaluable in optimising the uptake of telehealth use in DFD.

**Methods:**

This exploratory, descriptive qualitative study employed one-on-one, semi-structured interviews with health professionals who utilised the service. Thematic analysis using an essentialist inductive approach was employed.

**Results:**

Participants included 14 rural and remote health professionals; 2 general practitioners, 2 nurses, 1 Aboriginal Health Practitioner, and 9 podiatrists. In addition, 2 metropolitan-based TFS staff were interviewed. Five key themes were identified. ‘Patients have reduced travel burden’ included that telehealth enabled Indigenous patients to stay on country. ‘Patients had increased psychosocial support’ covered the benefits of having health professionals who knew the patient present in consults. ‘Improved access’ incorporated how telehealth improved interprofessional relationship building and communication.

‘Technological and equipment challenges’ highlighted that poor network connectivity and poor access to equipment to conduct telehealth consults in rural areas were barriers. The last theme,’Lack of service communication to rural health professionals’, highlighted the need for communication around service details.

**Conclusion:**

Telehealth is a valuable tool that can improve access to treatment for rural and remote Indigenous DFD patients. While this has the potential to improve DFD outcomes, empirical data is required to confirm outcomes. Considering the advantages of telehealth and rural staff shortages, there is an urgent need for investment in improved equipment and processes and an understanding of the training needs of the health care workforce to support the use of telehealth in DFD management.

## Background

Diabetes-related foot disease (DFD), which may be defined as ulceration, infection, ischaemia, or neuro-arthropathy of the foot [1], is a common complication in people with diabetes mellitus. DFD is one of the most prevalent causes of morbidity and hospitalisation and accounts for up to 75% of lower-extremity amputations globally [2–5]. By international standards, Australia has a high incidence of DFD-related hospitalisation and amputation [6, 7].

Diabetes-related foot ulcers (DFU) are often the precipitating event in major amputations [8]. Early identification of wounds and specialised multidisciplinary management can reduce amputation rates by 39–56% [9, 10]. Further, once a DFU is healed it is important to closely monitor patients as the risk of recurrence is up to 40% in the first year after resolution, 60% within 3 years, and 56% within 5 years [11]. 

Rural and remote communities are disproportionately affected by DFD [12]. Aboriginal and Torres Strait Islander populations, who have a high prevalence of diabetes, DFD, and major and minor lower limb amputation, are predominantly located in these areas [13, 14]. Central Australia (a large sparsely populated area of South Australia and the Northern Territory) is reported to have the highest rate of amputation in Australia, with Aboriginal Australians, particularly females and those undergoing renal dialysis, being disproportionately represented [15]. The vast geographical spread of Australia’s population and low population density is a substantial barrier to providing or accessing health services, particularly multidisciplinary and specialist services [16]. This limited access to care expertise may explain why Aboriginal and Torres Strait Islander and very remote areas in Australia have higher amputation rates than major cities or inner regional areas [17],.

Telehealth has been advocated as a strategy to improve equity of access to health care and timely access to specialist care in rural and remote communities [18]. In fact, research suggests that telehealth can promote timely diagnosis and treatment of DFUs to improve a patient’s quality of life and support the delivery of specialised foot care to rural communities [19]. Beyond improving patient access, telehealth has the ability to enhance team-based care by improving communication and collaboration between health practitioners [20].

The current literature describes various types of telehealth including telephone consults, photography, and video conferencing [21], with video conferencing providing the benefit of enabling real-time clinical observational assessment and supporting patient-practitioner relationships and rapport through visual cues [22, 23]. Clinical assessment of DFUs involves observational assessment of wound dimensions and clinical signs of infection [24]. Therefore, it is important for telehealth devices to enable clear observations of DFUs in diabetes care. While some applications have been developed for telehealth diabetes foot examination, very little has been implemented in practice [25, 26].

A recent systematic review of the clinical outcomes using telehealth for the diabetes-related foot complications found that audio/video/online communication approaches show similar healing efficacy for DFU as outpatient treatment [26]. This review identified continuity of care, adequate training of nurses, and nurses having good skills in wound management, as essential elements in the care of DFD [26]. However, most studies in this review were European. To our knowledge, little research has explored the use of real-time telehealth models involving podiatrists and vascular surgeons in the management of DFD.

Key barriers to the successful uptake of telehealth in any aspect of health in Australia include lack of staff acceptance, costs to patients and providers, technical issues, concerns about loss of continuity of care, patient confidentiality, privacy due to data security issues, and concerns over added workload by health professionals [21, 27–29]. Current literature suggests that successful implementation of telehealth requires access to user-friendly and reliable equipment, as well as adequate staff training and support [29]. Investigation into practitioner experiences of telehealth in the Australian context could be invaluable in understanding the needs of health practitioners in the implementation of telehealth services in DFD.

A large South Australian metropolitan hospital Vascular Surgery and Podiatry clinic has recently established a real-time video-based telehealth foot service (TFS) as a strategy to improve outcomes in rural and remote Aboriginal communities’ access to specialist DFD services. A 0.6 full-time equivalent (FTE) senior podiatrist was employed to facilitate all appointments as well as a 0.4 FTE Aboriginal Health Practitioner (AHP) to assist with cultural safety for Indigenous clients.

The TFS was co-designed through community engagement. Consultation was undertaken with three key Aboriginal Controlled Community Health Organisations (ACCHO’s) and the Northern Adelaide Area Local Health Network Aboriginal Consumer Reference Group. The appropriateness of the research method was arbitrated by the SA Aboriginal Health Research Ethics Committee. Funding for the telehealth program and this research were subject to continual approval by the Aboriginal and Torres Strait Islander Diabetes-Related Foot Complications Expert Advisory Committee.

All rural and remote health professionals in South Australia and the New South Wales border region of Broken Hill could refer to the TFS. Referrals were triaged according to the International Working Group on the Diabetic Foot guidelines (2019) and national evidence-based guidelines on prevention, identification, and management of foot complications in diabetes [30, 31]. Group consultation was scheduled as needed with a Vascular Surgeon and other specialists, including Infectious Diseases, Endocrinology, and Orthotics and Prosthetics. The pilot project ran for just over 18 months, from 5 November 2020 through to 30 June 2022.

To date, the use of real-time video-based telehealth for delivering DFD management in rural and remote Australia has not been formally examined. This study aimed to gain insight into the experiences of rural and remote health professionals utilising a newly established TFS. An understanding of health practitioners’ experiences of telehealth in the multidisciplinary management of DFD can highlight how clinicians can be supported in the use of telehealth to improve client outcomes.

## Methods

### Research design

This was an exploratory, descriptive qualitative study which employed one-on-one, semi-structured interviews to elicit rich descriptions of the feelings, opinions, and experiences of health practitioners who utilised the newly established TFS. An essentialist inductive approach was employed, meaning themes were derived from the data rather than pre-defined theory.

### Sample

Non-probability convenience sampling was used where rural and remote health professionals who had utilised the TFS were invited to participate by email. In addition, the TFS metropolitan-based podiatrist and Aboriginal Health Practitioner (AHP) employed to facilitate the TFS consults were interviewed. The email invitation included an information sheet and consent form. Participants’ written or verbal informed consent was gained before interviews commenced.

### Data collection

Individual interviews were scheduled at a convenient time for the participant and conducted either by phone, Zoom, or Microsoft Teams from 17 March 2021 to 16 June 2022. The interviews were conducted by the lead researcher (KG), a female academic who has tertiary qualifications in podiatry and psychology, a PhD, experience in high-risk foot management, and experience and training in qualitative analysis. To establish rapport with each interviewee prior to the interviews, KG disclosed her experience in podiatry and working in rural health. An open approach was used, where participants were asked to describe their experiences with telehealth. Follow-up questions, clarification, and probing were used to gain depth of recounted experiences [32]. Interviews with rural and remote health professionals lasted between 8 and 32 min and the joint interview with the two TFS staff based at the metropolitan hospital lasted for 58 min. All interviews were audio recorded with permission for analysis. Interviews were transcribed using an online transcription service and the research assistant (CMS) listened to each interview to ensure the quality of the transcription, including that the words transcribed were verbatim, and to familiarise herself with the participant’s data. Interviews were continued until no new information arose indicating data saturation had been reached [33]. Due to time constraints, participants were not provided with transcripts for comment prior to data analysis.

### Data analysis

Transcripts were de-identified and imported into Microsoft® Excel. Interviews were analysed using a six-phase thematic analysis technique described by Braun and Clarke (2016) [34] by the lead researcher (KG) and the research assistant (CMS). Three interviews were also analysed by a research assistant (EO) for rigour in the analysis process. Both research assistants are female and hold degrees in allied health and have experience with qualitative research. Emerging themes were discussed and refined with an experienced qualitative researcher (KMG), a clinical psychologist with extensive qualitative research experience, throughout the analysis process for clarity and rigour.

Each transcript was initially read separately by two researchers (KG and CMS) to familiarise themselves with the data. Codes presenting a unique idea which related to the research question were identified from the transcript and arranged in an Excel spreadsheet. Codes presenting a similar idea were grouped into themes and reviewed against the coded transcript to check that they accurately reflected the participant’s experience. The two researchers (KG and CMS) met regularly throughout the analysis to discuss their interpretation of the transcript and compare codes and themes to ensure similar ideas were identified. Once all transcripts had been coded and themes identified, themes presenting a similar meaning were grouped to create a list of key themes which portrayed the main ideas from all transcripts. The themes were then reviewed, clarified, and defined so that each theme had a clear and descriptive name.

### Trustworthiness and rigour

Inviting participants from a range of staffing levels (e.g. early career to clinical leads), settings (e.g. health services, hospitals), disciplines (e.g. nursing, medicine, podiatry), and geographic locations minimised bias and enhanced transferability. Dependability was increased by three authors coding the same transcript, the consistency of coding was checked, and the themes amended to ensure consistency. Finally, four meetings were held with the four authors involved in data analysis (KG, CMS, EO, & KMG) to review and discuss coding and development of themes which improved the confirmability of results. Findings were reported in accordance with the consolidated criteria for reporting qualitative research (COREQ) [35]. In addition to being interviewed for this study, the AHP had regular meetings to provide mentoring and feedback on the project with the researcher KG and read the final analysis of the results.

Ethics approval was obtained from The University of South Australia [approval 203786], Central Adelaide Local Health Network [approval 14286], the University of Adelaide [approval 35336], and SA Aboriginal Health Research Ethics Committee [approval 04–20-915].

## Results

Of the 25 rural and remote health professionals were invited to participate, 15 consented to an interview. One participant was excluded due to poor interview recording quality, resulting in a total sample of 14, including 2 general practitioners, 2 nurses, 1 Aboriginal Health Practitioner, and 9 podiatrists. The majority were female (*n* = 11) with a mean practice duration of 7.4 years (range 1 to 22 years). The geographical remoteness of the sites where participants’ health services were located was classified using the Modified Monash Model [36]. The sites involved in this study ranged from Modified Monash (MM) categories 3 through to 7, with MM3 being large rural towns and MM7 very remote communities [37].

Analysis of the interviews elicited five key themes (see Table 1). Each theme and associated subthemes are described below including example quotes from participants.

**Table 1:** Themes and subthemes

Patients have reduced travel burden	
Patients have increased psychosocial support	
Improved access	Assists with triage and rapid access to specialist services for patients
	The service was inviting to use as staff were accommodating, appointments were easy to access, and communication was enhanced
	Improved access to professional support, enhanced networking, and broadened skill set for rural clinicians which improved access to services for patients
Technological and equipment challenges	Reduced network connectivity in rural areas and poor platform performance
	Equipment unreliable or unsuitable
Lack of service communication to rural health professionals	Detail of the service’s existence, equipment required, and training
	Consult processes and making appointments
	Funding arrangements

### Research context

In the interviews, participants reinforced the premise of establishing the TFS, reflecting that rural and remote areas had limited access to specialised medical services. In some larger rural towns, appointments with general practitioners were difficult to access and if specialist services were available, they were provided by visiting health professionals. Remote towns may have nurses employed at a medical facility with only visiting GP and allied health services.

## Theme 1: Patients have reduced travel burden

Rural health professionals recognised the advantages of telehealth in reducing the need for patients to travel into metropolitan areas to access DFD services, particularly for Indigenous patients:

Unfortunately, one of the barriers with this patient is transport. So he already has a previous amputation in the other leg and there is no way to transport him to the clinic. (Participant 6).

A lot of people would prefer to stay on Country if they can be closer to home, they're probably going to take up the service a lot more. (Participant 7)

The reduced time away from home allowed patients to continue participating in their family and work commitments. For example, being able to remain in their rural town improved the opportunities to have a support person attend the consult with the patient. This was particularly helpful if a patient or the support person had barriers with communication or travel:

I had a gentleman that his wife's going through cancer treatment, so she can't travel So, for her to be able to be a part of it, it's beneficial for their whole family really. (Participant 6)

Some patients were so reluctant to travel to a metropolitan hospital that they may choose not to engage in treatment:

I think definitely the lack of having to drive down, the lack of that time for patients is a massive plus. And, I've probably had a couple where the way I've explained it has been like, "Okay, I'll agree to do the telehealth," versus, "I don't want to go see a specialist. I don't want to go to Adelaide." Whereas then giving them that option, it's kind of been like, "Oh, okay. I actually will take their advice and their input." (Participant 5)

One positive for patients is the ability for medical investigations and treatment planning to be conducted locally, enabling treatment decisions and planning to be completed while the patient stays at home and streamlined admission to hospital. As a result, patients could spend less time in hospital having investigations, delay admission to hospital, or be discharged from hospital sooner as follow up could be conducted via telehealth. This extra time enabled patients to plan for their hospital stay and health services to be responsive to patients needs such as organising resources to support the patient participating in the treatment plan:

I think it's a much more streamlined process, … it means the patient knows a bit more about what their prognosis might be or what the next steps are or whether they need debridement or amputation or antibiotics or monitoring or lots of dressings. All that can be done without them necessarily having to be physically seen down there in an urgent way … I think it actually helps them [the patient] to probably understand why they might need to do that [go to the metropolitan hospital]. And the timeframe allows them to prepare, live with family, and for us to get our resources together, to help them to get to [the metropolitan hospital], rather than it being more of a crisis situation and a bit more of a rush or a hospital transfer. So, I think it does improve engagement and it allows it to be done more as an outpatient than an inpatient. (Participant 9)

However, there was an increased time burden on point-of-care health professionals who reported requiring longer consult times:

The patient will nod along and be like, "Yep, yep. I understand that," and then afterwards, if I go, "Oh, so that all makes sense?" then they're like, "No." And then I do have to explain it. (Participant 5)

## Theme 2: Patients have increased psychosocial support

Being in a familiar environment with trusted, culturally supportive health professionals can be improve Indigenous patients sense of safety and engagement in treatment:

She being told that it's different to that and that we're all working closer together and that there is that open line of communication that, I think knowing that she has cultural and emotional and mental health aspects of trust in our service, knowing that we were going to be involved in that and be able to advocate for her and having our Aboriginal Health Practitioner in there with her, I think she found that very, very helpful. I think she found a lot less confronting and a lot of safer environment which means she felt more able to engage with that too. (Participant 13)

They're already going to their health services [ACCHO’s] anyhow for a whole range of reasons. And it's close access, they know the people, it's comfortable, it's safe ... And I think they felt more comfortable. (Metropolitan TFS staff)

A rural health professional who has a history with a patient may understand the patient’s level of health literacy and notice cues that the patient requires further clarification during a consult:

The patient can talk, but I find [it] comes with the health literacy of the patients, we act as an intermediary anyway. So sometimes the patients need that clinician interface locally to assist what's been discussed on the telehealth. (Participant 9)

The metropolitan TFS staff valued the medical and psychosocial background that rural health professionals provided about the patient:

We do very much value the knowledge the clinicians have on the other end, because they know a lot about what's going on in that patient's life and their input is very important. (Metropolitan TFS staff)Involving the AHP in the metropolitan TFS improved the identification of indigenous patients’ broader social determinants of health which may be impacting their health outcomes:

More of the social and emotional well-being stuff, is the role that I play with it, and not so much, very little clinical stuff … There's no point telling them what they need to do clinically, without addressing the social and emotional well-being side of things. Because if they've got other stuff going on, whatever it is … Yeah, nowhere to sleep, no food to eat. You're not going to care about your foot, especially if you can't feel it … until you've sorted out all the other stuff. A lot of the time people are in survival mode almost, so they do require a lot of extra support. (Metropolitan TSF staff)

The metropolitan TFS staff reported that communication in telehealth appointments was enhanced:

The patient talks a lot more in telehealth than in outpatient clinics. They ask a lot of questions. They're really involved a lot of the time, and want to know more information, and often share more information about themselves as well. (Metropolitan TFS staff)

The metropolitan TFS staff felt this may be because the telehealth environment is more conducive to discussion than the busy multidisciplinary clinics:

The foot clinics, they are quite crazy, and you've got lots of people jumping in and looking at you, and not really having the time, as much as people would love to, but to really talk and get to know the person, and understand what's going on. (Metropolitan TSF staff)

The metropolitan TFS staff felt that having dedicated staff for this project improved communication. These staff built relationships over time with patients, which included meeting patients face-to-face when they were admitted. The TFS staff felt that developing these relationships built trust and improved engagement with treatment:

Because we made a point of seeing every patient that gets the high-risk stuff … And the ones that we haven't met, we've been able to have a yarn with, and get to know them, and feel comfortable with each other. And they feel more safe being there. (Metropolitan TFS staff)

An additional factor that may improve communication is the AHP improving cultural safety via educating non-Indigenous staff:

… I think [the AHP] has taught me and made me very much aware of the mistrust that's in the [large metropolitan hospital] people don't trust you straight away. They are very, you know, you're a white face, you're in the [large metropolitan hospital], you can feel that they don't want to tell you what's going on. They don't want to listen to what you say. They don't want to engage. (Metropolitan TFS staff)

## Theme 3: Improved access

### Assists with triage and rapid access to specialist services for patients

Participants found the TFS made it easier to escalate patients:

Trying to escalate a patient then [before the TFS was established] was quite difficult. While now with telehealth, their telehealth is acting as that escalation and support mechanism. (Participant 4)

Most participants valued that the metropolitan TFS team assisted the rural team to triage patients by helping assess the appropriateness and urgency of referrals. Moreover, the TFS helped improve the speed of access to specialist services through bypassing the traditional referral pathways, which often involve lengthy wait times:

I think it's made access to high-risk foot clinics much more streamlined and easier to be honest. Because it means that certainly the tertiary centres can get a quicker access to that patient. And, I must say, [the metropolitan hospital] has been very good at facilitating our access to high-risk podiatrist services and the vascular and orthopaedics … and then we can make a decision at that first telehealth as to the urgency. (Participant 9)

Participants provided examples of where rapid access to specialist clinics had improved patient outcomes:

But having that input of the people higher above us, definitely... That is the reason why this patient possibly isn't going to lose his foot. (Participant 13)

Two participants commented that telehealth could improve equity of access to care, particularly for remote and indigenous people:

Telehealth, I think it's an amazing solution to try and get that increase in equitable healthcare across the board for especially our rural consumers … I think in terms of closing that gap, it's definitely helped. (Participant 4)

Participants recognised that timely triage has the capacity to create efficiencies and reduce strain on the health system:

Because the biggest issue we've had here is, obviously the [metropolitan hospital] is having capacity issues at the moment, so we've got patients that are inpatients, that are really poorly, but can't be transferred and have been offered outpatient appointments. And for someone that's bedbound it just doesn't seem fair on the health system and on the patient to cover the cost of an ambulance transport down to [the metropolitan town]. And then them having to sit in the transport only for an outpatient appointment and then to come back… So that's been really good. Just being able to be like, “That’s fine, it can wait'' or, ''No, they need to come now.'' (Participant 1)

Despite the improved access to specialist services, participants acknowledged the limitations of telehealth as some services can only be delivered face-to-face:

We've still got those barriers ... We don't do total contact casting. So you can get that podiatry input but, then the actual hands-on stuff was stuck. (Participant 2)

### The service was inviting to use as staff were accommodating, appointments were easy to access, and communication was enhanced

Rural health professionals felt invited to utilise the TFS, and most reported feeling supported by the TFS staff and comfortable asking for advice:

if in doubt, I can basically just call the on-call phone and just say, "Look, I'm concerned about this job on telehealth," or whatever, and have a look. (Participant 5)

Because the TFS podiatrist carries the on-call phone, when participants phoned to make an appointment, they typically spoke directly with the TFS staff and most mentioned the helpfulness of having an accommodating and flexible on-call service:

[The podiatrist] was awesome. She just owned it. I might add, it was at 2:30 on Thursday before Easter and she was going to be leaving in an hour for Easter. She was switched on. She got it all done. (Participant 10)

In addition, participants appreciated that the TFS provided easy to access emergency appointment.

The metropolitan TFS staff reported that patients also utilised the ability to directly contact the TFS staff to talk about treatment, psychosocial issues, and to alter appointments. The ability for Indigenous clients to easily change appointments led to increased attendance rates (> 90%).

Having both the patient and rural and metropolitan health professionals present enhanced communication, by assisting in clarity of the consultation content between parties. In addition, telehealth helped overcome delays and inadequacies in clinical handover between health professionals:

[I] desperately think that more services should be providing the ability of this kind of care because it's the ability for clients to have clinic staff be present with them so that everyone knows what's going on and the communication structure that they've had. … Hospital specialists don’t always send us reports, so this is better. And getting those pretty much straight away is fantastic because it means that we're all on the same page. (Participant 12)

Further, most participants found this aspect of telehealth often reduced some of the burden of traditional paperwork and follow up required between rural and metropolitan sites:

It also takes away from the need to actually then communicate via letter or via an email afterwards. (Participant 11)

### Improved access to professional support, enhanced networking, and broadened skill set for rural clinicians which improved access to services for patients

Telehealth has the capacity to help reduce some of the impact of professional isolation that rural professionals can experience:

It was good just to have input from another hospital … it can be quite isolating. And then for us, it's just connections with other people, experts that know what they're doing. (Participant 10)

Participants enjoyed seeing the faces of the people they had been communicating with in written forms:

Put a vascular surgeon's name and face together as well … I guess it kind of makes things a bit more comfortable, I think, and a bit less formal too. (Participant 12)

Participants felt supported and comfortable asking for advice or a second opinion with clinically challenging cases:

It was good to have that support too, from the high-risk foot services to make sure that our current treatment plan, we're on the right track with how we were managing things for the client. (Participant 11)

I think that they're [the TFS team] absolutely wonderful. And even just having another set of eyes, they tell you like, ''Oh try this.'' And then you're like, ''Oh yeah, I didn't think of that.'' (Participant 1)

This improved accessibility increased health professional networks and supported rural health professionals’ clinical skill development:

I feel like I can ask her a clinical question, not second-guessing what you're saying, but like, ''I just wonder what your reasoning was behind that''… So being able to learn someone else's clinical reasoning has been good. (Participant 1)

One participant found having a second health professional support their treatment plan supported treatment concordance:

Sometimes just having that metro voice really helps getting the patient to adhere to their offloading plan or to take their antibiotics properly or look after their sugars or whatever it might be. (Participant 4)

Participants particularly enjoyed the learning experience of being in a consultation with vascular surgeons:

If vascular link in, yeah, there's definitely stuff to be learned from some of those conversations that they have with the client. (Participant 14)

However, almost half of the participants expressed that they were disappointed when a vascular surgeon was not present at the telehealth consultation as they felt having access to vascular surgeons was more beneficial to the patient:

It would be nice to have the vascular there all the time. (Participant 5)

You don't often see vascular, it's kind of just with podiatry on the other end, … But when you're wanting something more and that's not there on that particular day, that can then be a bit of a barrier into getting the right care because it's kind of just doubling up on the service that the client's already getting. (Participant 14)

The establishment of positive interprofessional relationships between rural and metro teams supported timely and responsive escalation of patient care when required:

I think the users on the other end, they've worked in country, or they've got a bit wider understanding about if we're reaching out for assistance, we really need that assistance now. And they're quite responsive, which is good. But my own personal experience is it has improved collaboration between the two. (Participant 4)

Both the TFS and rural health professionals felt that over time, trust between the teams improved which supported the patient’s continuity of care:

As it went on, the relationship got stronger. And trust, telehealth is here to work together to make sure the patient gets the care that they need. (Metropolitan TFS staff)

Not only rural-metropolitan relationships were built, but the TFS also helped establish relationships within the metropolitan hospital, for example between the Aboriginal and Torres Strait Islander Health and Wellbeing Hub and the high risk foot service:

Before TFS the Aboriginal hub did not know podiatry existed at [the metropolitan hospital], now we all know each other by name. We're getting actual referrals from the hub. (MetropolitanTFS staff)

It was reported that telehealth had assisted in building relationship between some ACCHO’s and local health networks (LHN):

The Aboriginal community-controlled sites don't talk to the LHNs a lot of the time. … Telehealth has been a way to bring them together in some areas. (Metropolitan TFS staff)

## Theme 4: Technological and equipment challenges

### Reduced network connectivity in rural areas and poor platform performance

Rural and remote areas were often technologically disadvantaged, reporting poor internet connectivity, only 3G mobile data connection, and poor sound and picture quality:

We don't have Wi-Fi access. So it's trying to have a big, long internet cord connected through or where, if we're out in the region, the service isn't that great. (Participant 14)

To compound these difficulties, participants reported frequent challenges using the Healthdirect video call platform established by the federal government for telehealth consultations. These issues increased time pressure for health professionals. Rural participants were creative in working around many technological issues, overcoming the challenges of Healthdirect, Wi-Fi “black spots”, or network connectivity issues by using their personal phones to make phone calls, using FaceTime, and sending photographs through text messaging to facilitate the telehealth consultation:

Healthdirect [is] failing as a platform. And you end up having to do the rest of the consult via FaceTime or just on a telephone call. (Participant 4)

### Unsuitable or unreliable equipment

Some participants reported having old treatment chairs that were not mobile and TV screens fixed to the wall, resulting in poor ability to position equipment for a telehealth consultation. Many rural foot services did not have access to reliable equipment, or the specialised equipment required for telehealth:

The camera on the computer decided not to work... so unfortunately we ended up just talking on the phone. (Participant 7)

In [a rural town], we weren't able to get a laptop. We would borrow one of the iPads from telerehab. (Participant 4)

I find that telehealth's a little challenging in terms of being able to show you the patient's foot with a camera on a fixed computer. (Participant 9)

Several staff preferred using photos from a high-quality phone for telehealth consultations as it provided better visual quality of an ulcer compared to the available equipment. Although participants had different preferences for the type of device used, most agreed on the value of manoeuvring the device to the ulcer during consultations. Two participants had access to small handheld cameras able to capture high quality images, which they believed was important for accurate assessment of the wound.

I guess if it’s a poor-quality image of the wound, the podiatrist on the other end can’t even really see… if you’ve got that Logitech camera, it’s good. But if not, if you’re trying to do it off your phone or your tablet it’s not as effective, not as clear (Participant 8).

Additional equipment related issues raised in interviews were the safety of multiple electrical cords, trying to follow appropriate infection control procedures while using equipment, requiring longer consultation times to allow for equipment set up and treatment before or after the telehealth consultation.

## Theme 5: Lack of service communication to rural health professionals

### The services existence, equipment required, and training

One remote locum health professional was unaware the service existed, and suggested strategies were needed to ensure rural health professionals knew of the TFS:

I think if there's better awareness that this resource is available for us to then have it as a resource… Because I actually didn't know this existed. (Participant 13)

Some participants thought details about the telehealth service and the equipment required by rural health services could be more broadly and clearly communicated to rural health services:

When the service was set up, they kind of just said, this is what we're doing, but didn't really give us much of an idea of what we would need. (Participant 14)

Some rural participants felt that the platform was user friendly, but some found logging into the Healthdirect platform a challenge and thought that they had not been provided with the appropriate training prior to its use:

It took me a little while to actually get set up … So I had to do a bit of phone calls and emails and running around to try and find out how I actually start the whole process. (Participant 11)

### Processes for consultations and making appointments

Several participants felt there could be improved communication about what medical investigations were required prior to the consultation and suggested having a protocol outlining telehealth consultation could be of benefit:

Having something in writing… like a health pathways kind of document that says you'd like these particular tests before you see a patient… these particular images at these particular angles, they're always kind of helpful things for our end… And also who you would like to be on the telehealth that you'd find helpful. (Participant 9)

Three rural participants found there was not enough clarity around the process of making appointments:

It's sometimes unclear when booking the telehealth as to whose responsibility, who does what, … It makes sense for the local team to call and book the patient in because they know the directions, how to get there, where the car park is and all those sorts of things. So again, probably just a bit of clarity from metro to country about who does what in terms of booking that appointment would sometimes be a bit better. (Participant 4)

Two participants felt the TFS staff did not understand some of the difficulties for rural and remote health professionals and patients, such as travel between sites:

They're [the metropolitan staff] probably not very flexible with time… So, that means that we have to move other people around. Or, another thing, probably because they're metro that we don't consider some of the regional aspects. So they might be like, "Oh, I want to see so and so at Friday, at 10 o'clock," but they're not located in [the major regional town]. They might be in [a small rural town] four hours away. So, it's probably like they almost should be revolving around when we are seeing the client. (Participant 8)

It's probably more difficult from our end just because our appointments are often booked up. We don't have necessarily time kept aside each day for if these requests come through for a telehealth consult. (Participant 14)

### Funding arrangements

Participants felt that it was important to communicate information about telehealth service funding arrangements:

If it's something that they want to continue on long term, it's probably more looking at funding, how it's getting funded. Who's getting the funding. Because obviously it's great to have their numbers grow, but if our FTE's not growing out in the region, we don't then have the capacity to always be providing those services and that sort of thing. (Participant 14)

One participant found patients were confused about why they had to pay for some services in the rural or remote locations, but they did not have to pay at the metropolitan based TFS:

“They have to pay for their offloading, all of that. And then they go down to the [metropolitan hospital] and they don't have to do that. And then the patient gets, ''Well, who should I be going with?'' And that kind of thing. (Participant 1)

One participant thought some of the uncertainty about funding may originate from how different local health networks (LHNs) interpret the funding arrangements:

Depending on what episode type we see the patient under depends on how much they're charged for that appointment. And so if we're seeing someone under a Commonwealth Home Support Package episode, they're charged in the number of minutes that they're in the chair for, while if we're seeing someone under community health, it's just per presentation. And then sometimes we see them under an outpatient episode where it's free.

So again, different consumers are having to contribute different amounts depending on where they are and what episode type they fall under too. So I guess in terms of that continuity, it's not there from a regional LHN point of view because we're now six regional LHNs. And they all interpret the rules around telehealth a little bit differently. And all do it differently. (Participant 4)

Despite the challenges, rural health professionals could see the benefits to patients of the telehealth service and were grateful to have the service:

I think that's [telehealth] probably the best thing that's come out of COVID, that tertiary centres and private specialists have finally embraced telehealth. (Participant 9)

## Discussion

This qualitative exploration of health professionals’ experience using a real-time video-based telehealth foot service (TFS) for DFD management has provided some valuable insights. Five key themes were elicited: ‘Patients have reduced travel burden’, ‘patients have increased psychosocial support’, ‘Improved Access’, ‘Technological and equipment challenges’, and ‘Lack of service communication to rural health professionals’. There was overwhelming support from health professionals that telehealth has the capacity to improve access to services and engagement in treatment for Indigenous DFD patients. In addition, it is a valuable tool for triaging patients and building interprofessional relationships. However, some barriers exist such as technological poverty, lack of clarity around processes, the need for longer consultations, and understanding of the difficulty for rural and remote health professionals in participating.

This research supports previous findings that telehealth reduces travel burden for rural patients [22]. Moreover, telehealth provided opportunities to receive care in a safe and familiar environment [22, 38] improved ability to maintain family and work commitments, and improved attention to the patients’ understanding and health literacy. As telehealth can delay patients’ admission to hospital, discussions around complex clinical decisions are being included in telehealth consultations. In the authors opinion, having these challenging conversations in a familiar environment, surrounded by health professionals the client knows may offer some advantages compared to the busy high-risk foot clinic with many health practitioners, who the patient may not know. The impact for rural practitioners was that these discussions required longer appointment times on top of an already heavy workload. However, the additional time at home allowed for services to be responsive to patients’ needs by initiating support such as assistance with travel. Our findings suggest that a model of care which embed an AHP and where Indigenous patients can be seen at their local ACCHO or with their usual health provider may improve cultural safety, engagement, and attendance rates. Holistic person-centred approaches to health, which address more than pathology but include elements such as social determinants of health, connectivity to country, and cultural support are well documented as key in Aboriginal health management [39]. A valuable by-product noted in our findings was that the inclusion of an AHP provided opportunities for on-the-job exposure to cultural sensitivity as an incidental outcome. Improving engagement in DFD management is particularly important the Aboriginal population in Australia who have been found to be up to 38 times more likely to undergo an amputation than non-Aboriginal Australains [14].

Feedback validated the use of a telehealth on-call phone service to provide flexible access, rather than only fixed appointments and referral pathways. The improved attendance at the TFS service highlights a systemic issue with policy that can impact Indigenous people. Indigenous people have cultural requirements that may mean appointments cannot be attended at short notice. Multiple missed appointments may result in patients being discharged from a service, placing them at great disadvantage to access care in the future. The authors hypothesise this improved attendance was a result of a combination of continuity of care provided by the TFS team and the ability to directly contact the TFS staff to change appointments.

The findings from this current study illustrate that this model of care, with AHPs, and rural and remote health professionals in the consultation with the patient has several advantages, specifically around improving patients’ access to services and health professionals’ access to each other. For example, triage was improved by having the rural and remote health professionals to offer context, history, and psychosocial information in addition to the metropolitan specialists’ expertise. This process helped prioritise the use of specialist services which may reduce some burden on metropolitan hospitals. Further, when needed the TFS provided rapid access to specialist services which likely improved patients’ outcomes; however, empirical evidence is required to quantify this. Traditionally, specialist referral pathways can take some time as it involves a GP consultation for referral, which can be very difficult to access, then waiting for a specialist appointment to become available.

An interesting point raised was that communication between the tertiary hospitals to rural practitioners following the traditional pathways was often inadequate. Health professionals highlighted that this model of care, with all health professionals present in the telehealth consultation, improved the speed and clarity of communication and avoided delays created by waiting for reports. Furthermore, this model reduced some of the post-consultation administrative paperwork.

Rural and remote health professionals enjoyed being able to put a face to the name of the metropolitan staff they refer to. Our findings support that the building of interprofessional relationships facilitated by telehealth [20] may improve collaboration between metropolitan and rural sites and continuity of patient care. Interprofessional collaboration is thought to optimise patient care, facilitate continuity of care and access to care, and therefore improve patient outcomes [40]. Further, our findings suggest exposure to other health professionals in this multidisciplinary telehealth model may increase rural health professionals job satisfaction and therefore may be a valuable tool in retaining rural staff.

The accommodating attitude of the TFS facilitated relationship building, which aligns with previous findings of the crucial role ‘champions’ play in the success of establishing new services. In addition to promoting the service, champions play an important role in relationship building [41]. Participants found the TFS staff accommodating and supportive, which appeared to improve their likelihood of referring to the TFS. Our findings suggest relationships on all levels were an important component of establishing the TFS service, including between patient and health care provider, between professionals, and between health care sites. However, while building relationships can improve access to services for patients, they take time to establish [42].

A major barrier to effective TFS consultations was the technological poverty experienced in rural and remote Australia, particularly poor internet connection, poor access to appropriate equipment, inadequate training, and the poor performance of the Healthdirect platform [21, 28]. Although some of these problems have been highlighted in past research, strategies to address them are yet to be widely translated into practice. Some services struggled to access basic equipment to capture images of wounds, while a few had recently purchased portable cameras for the sole use of telehealth and reported these were very effective at capturing high-quality, usable images of ulcers. Importantly for podiatry in particular, standardised video call setups are frequently inimical to illustrating foot wounds. Additional concerns included the safety of cords for technological devices in treatment areas and maintaining infection control while using telehealth equipment.

Concerningly, health professionals reported frequently having to resort to using their personal mobile phones and social media platforms to overcome connectivity and platform inadequacies. This has also been identified as common practice in dermatology [43]. The risks associated with the use of mobile phones to produce and store medical images include consent, privacy breaches, insecure data storage, and physician or institution liability as well as the potential for security breaches if electronic mobile devices are hacked, lost, or stolen.

### Limitations

This study was conducted in South Australia and the NSW border region of Broken Hill and thus caution should be taken when generalising to other settings. We sampled clinicians from a geographically broad variety of health services and hospitals, for the findings to best reflect the experiences of people working in different health services across various locations in South Australia. Although we set out to recruit a purposeful sample, most health professionals who participated were female, podiatrists, and non-Indigenous. Therefore, the authors potentially missed capturing some of the specific attitudes and experiences of males and other health professions, particularly those that identified as Indigenous.

### Recommendations and future research

Embedding holistic person-centred care approaches into busy, time poor multidisciplinary settings is a challenge. While the focus of this project was Indigenous patients, these findings should not be ignored for other telehealth services as a holistic approach to health care has been found to be beneficial in many settings [44]. However, with Aboriginal and Torres Strait Islander populations located mostly in areas with poor access to services and being at increased risk of amputation, future research should focus on the elements of telehealth service that can assist this community. To further our findings, examining the impact of continuity of health care team, methods to further improve communication and collaboration between the different health services, and ways of increasing flexibility of services to accommodate the need for Indigenous people to participate in cultural commitments could be explored.

A strong theme emerging from the interviews was that the TFS assisted with patient triage and streamlined referral processes. Notwithstanding this, health professionals voiced a need for a process-type document to outline investigations and equipment required for the TFS consultation. Efficient clinical governance clearly helps to avoid duplication of investigations and using health professionals time resourcefully.

Rural and remote health professionals thought information about the TFS could be disseminated more widely, to provide better knowledge that the service existed, outline processes for making appointments, and ensuring suitable equipment is available to use in consultations. In addition, our findings suggest exploring differences in how LHNs interpret, and access funding may be worthy of attention. If telehealth services are to expand, how the costs of the service and equipment will be adequately covered by rural and remote health services is an important consideration.

While it is well recognised that the requirements of communication in telehealth are different to those in face-to-face consultations, there is little research into the impact of communication in multidisciplinary telehealth consultations. Communication skills are essential for every consultation and can help avoid misunderstanding; however, there is a wide variability in the development of these skills among health professionals. Therefore, it is essential that communication on different platforms is covered in undergraduate training. Postgraduate workforce development and education opportunities should also be addressed. Research could further explore communication and therapeutic relationship-building on the telehealth platform.

Adding together our findings, those of past research, and the improved access to health services offered by telehealth, there is an urgent need for investment in purpose-specific, user-friendly, and reliable equipment that enables the easy capture of foot ulcers while maintaining a safe treatment environment which meets infection control standards [29] . In addition, realistic policies and adequate software resources are critical to ensure the protection of patients and practitioners using telehealth services [43]. Further, the development of best-practice principles for telehealth consultations should be established. These should take into consideration informed consent for images and disclosure of details about how the photographs will be used and stored, as well as how to ensure adequate safeguards for patients, health professionals, and services [45]. Finally, further research is needed to provide empirical evidence in DFD outcomes in telehealth.

## Conclusion

Our findings support that this model of telehealth improved access to specialist foot services for Indiginous DFD patients in rural and remote communities and therefore is an important tool in combating the high rate of DFD complications in this population. Moreover, continuity of staff, embedding an AHP in the service, and a service that enables the ability to easily change appointments may improve access and engagement with the at risk Indigenous population. The reduced travel burden on patients, improved psychosocial support for patients, and improved triage of DFD patients show potential to reduce cost burden on both patients and health services. In addition, telehealth may reduce some of the professional isolation experienced by rural and remote health professionals and that building interprofessional relationships may improve referral rates. However, significant efforts should be taken to address the digital divide, provide purpose specific equipment, and ensure sustainable funding. In addition, there is a critical need for research and metric information to assist the development of evidence-based best practices and policies, user-friendly processes, and to guide education requirements to support the new models of care offered by telehealth. Considering the rate of technological progress and rural staff shortages, telehealth shows promise as a valuable equity enhancing rural health tool.

## Data Availability

The datasets during and/or analysed during the current study available from the corresponding author on reasonable request.
